# Same-Day Magnetic Resonance-Guided Single-Fraction Stereotactic Body Radiation Therapy for Painful Non-Spine Bone Metastases – A Single-Center Study (“BONE SHOT”)

**DOI:** 10.1016/j.ctro.2025.100966

**Published:** 2025-04-27

**Authors:** Sebastian M. Christ, Eva-Maria Kretschmer, Michael Mayinger, Madalyne Day, Nienke Weitkamp, Amanda Kristina Moreira, Stefanie Ehrbar, Cäcilia S. Reiner, Marta Bogowicz, Lotte Wilke, Stephanie Tanadini-Lang, Nicolaus Andratschke, Helena I. Garcia Schüler, Matthias Guckenberger

**Affiliations:** a Department of Radiation Oncology, University Hospital Zurich and University of Zurich, Zurich, Switzerland; b Institute of Diagnostic and Interventional Radiology, University Hospital Zurich and University of Zurich, Zurich, Switzerland

**Keywords:** MR-Linac, SBRT, Bone metastases, Palliative radiotherapy

## Abstract

**Introduction and background:**

There is evidence for efficacy of high-dose single-fraction stereotactic body radiotherapy (SF-SBRT) for painful non-spine bone metastases (NSBMs). This study (“BONE SHOT”) assessed feasibility of same-day magnetic resonance-guided (MRg) planning and SF-SBRT delivery, recorded toxicity and assessed efficacy for treating metastatic patients with NSBMs.

**Materials and methods:**

Patients with painful (≥3/10 points on a 0–10 numeric rating scale (NRS) for pain) and radiologically confirmed NSBMs from solid organ malignancies were eligible for this prospectively acquired, single-center study. Patients received MRg-SF-SBRT via MR-Linac (ViewRay®) with same-day consultation, consent, planning and treatment. Drop-out rate, procedure times, acute toxicity and pain response were recorded.

**Results:**

Between June 2019 and June 2020, 13 patients with 15 NSBMs were treated per protocol. Mean patient age was 64 (range, 30–87) years; most common primary cancer was gastrointestinal malignancies (38.5 %); most commonly treated site was pelvis (53.3 %). All workflows were completed as planned. Median on-table time for contouring, planning and delivery was 65 (range, 57–112) minutes. Treatments were well tolerated; one patient developed “pain flair”; no grade ≥ 3 toxicities were registered. At one week following SBRT, overall and complete pain response rates were 73.3 % and 20.0 %, respectively, which evolved to 66.7 % and 53.3 % at four weeks after SBRT; median pre-treatment pain score was 6 points, which was reduced by a median of 5 points (P = 0.0028) at four weeks.

**Conclusion:**

The same-day MRg-SF-SBRT workflow for NSBMs was feasible, safe, and preliminary results indicate promising efficacy, warranting future trials investigating this intervention.

## Highlights

• High-dose single-fraction stereotactic body radiotherapy for painful non-spine bone metastases has shown to be efficacious.
• This study (“BONE SHOT”) assessed feasibility of same-day magnetic resonance-guided planning and SBRT delivery.
• All workflows were completed as planned; and treatments were well tolerated.
• Overall and complete pain response rates were 73.3% and 20.0%, which evolved to 66.7% and 46.6% at four weeks after SBRT.
• Same-day magnetic resonance-guided SBRT for non-spine bone metastases was feasible and safe.

## Nomenclature

BASEC: Business Administration System for Ethics Committees
BMs: Bone metastases
CR: Complete response
CT: Computer tomography
CTCAE: Common Terminology Criteria for Adverse Events
ECOG: Eastern Cooperative Oncology Group
GTV: Gross tumor volume
ICRU: International Commission on Radiation Units and Measurements
NRS: Numeric rating scale
MF: Multiple-fraction
MRg: Magnetic resonance guided
MRI: Magnetic resonance imaging
NSAR: Nonsteroidal anti-inflammatory drugs
NSBM: Non-spine bone metastasis
NSCLC: Non-small cell lung cancer
OARs: Organs at risk
PET: Positron emission tomography
PR: Partial response
PTV: Planning tumor volume
RCC: Renal cell carcinoma
RT: Radiation therapy
SBRT: Stereotactic body radiotherapy
SF: Single-fraction
OR: Overall response
QA: Quality assurance
QoL: Quality-of-life

## Introduction and background

Bone metastases (BMs) are common in cancer patients and can be the cause of debilitating pain [1]. Radiation therapy (RT) constitutes a well-established, palliative treatment modality for painful BMs [2]. Following the advent of new systemic therapies like targeted agents and immunotherapy, and advances in surgical and radiation technology, metastatic cancer patients can achieve better oncological outcomes [3]. With improved survival in metastatic cancer patients, durable symptom control, quality-of-life (QoL), and therapy convenience become even more important.

Various low-dose RT fractionation regimens such as 30 Gy/10fx, 20 Gy/5fx, and 8 Gy/1fx have been shown to be equieffective for palliating cancer-related bone pain with similar toxicity profiles [[4], [5], [6], [7]]. Yet as multi-fraction (MF) schedules achieves more durable response times than single-fraction (SF) ones, with the consequence of more frequent re-treatments after SF-RT as compared to MF-RT [[4], [5], [6], [7]]. In clinical routine, this often leads clinicians to opt for MF-RT in patients with painful BMs in good performance status. There is consequently room to further improve efficacy of RT with respect to pain response and durability of pain response.

Randomized trials on spine metastases have demonstrated effectiveness of stereotactic body radiotherapy (SBRT) for pain control and local tumor control, leading to current ESTRO and ASTRO guidelines that recommend SBRT for patients with limited spine metastases, particularly in cases of oligometastatic disease or when more precise targeting is needed to minimize damage to the spinal cord [8,9]. With the advent of SBRT, the adoption of dose-intensified hypofractionation schedules has transformed RT for many disease sites, and is increasingly studied and applied in the context of BMs [[10], [11], [12], [13]]. In 2024, *Guckenberger et al.* published the results of a trial comparing conventional radiation therapy with SBRT for spinal BMs, reporting an improved pain score at 6 months for patients treated with SBRT [10]. *Nguyen et al.* (2019) conducted a phase II randomized trial comparing SF-SBRT with MF-RT for predominantly non-spine bone metastases (NSBMs), and also reported more prolonged pain control in patients having received SF-SBRT with equal toxicity and QoL [14]. Several reviews elucidating the potential role of SBRT for BMs emphasize the more durable pain responses, while highlighting the need for additional prospective, randomized studies, especially for NSBMs [15,16]. A *meta*-analysis on SBRT for NSBMs by *Moraes et al.* (2023), which included the mainly available retrospective studies, concluded that SBRT is both efficacious and safe [17]. A recently published paper by *Burgess et al.* (2024) bundles the opinions on the principles and practice of SBRT for BMs by experts in the field. The publication stated the increasing use of SBRT for BMs, yet also noted that evidence for the adoption of SBRT for NSBMs remains weak [11]. However, SBRT has the limitation that its complexity is increased as compared to standard palliative radiotherapy, resulting in longer intervals between patient consultation, start of RT and response to RT.

MRI-guided online adaptive RT has the potential to take the benefits of SBRT and simultaneously eliminate its disadvantage of long preparatory and planning times. Simulation using the hybrid MRI-Linac technology may allow accurate target volume and organs-at-risk (OAR) definition and the online adaptive capabilities may allow for immediate same-session treatment. Such a rapid RT access and treatment strategy could have the potential to decrease the time interval between patient consultation, reduce the time until pain response, thereby improving QoL and patient compliance. We therefore conduced a prospectively acquired study to test the feasibility of same-day and single-session SBRT for painful NSBMs using an MRI-guided online adaptive workflow.

## Materials and methods

### Study design and objectives

This prospective study was designed as a single-center feasibility study and was conducted at the Department of Radiation Oncology at the University Hospital of Zurich (USZ). The primary endpoint of this study was to test feasibility of a same-day single-session MRg SF-SBRT procedure for NSBMs. Feasibility was assessed with the proportion of patients successfully treated using the same-day and single-session approach. Secondary endpoints were workflow procedure times, acute toxicity using the Common Terminology Criteria for Adverse Events (CTCAE) v5.0, and efficacy in the form of a positive pain response, which followed *Chow et al.*’s 2012 publication on the “Update of the International Consensus on Palliative Radiotherapy Endpoints for Future Clinical Trials in Bone Metastases” [18].

### Study inclusion and exclusion criteria

Patients were eligible for inclusion into this study if they had a histopathologic proven diagnosis of a metastatic solid malignancy, radiographic evidence of NSBMs on a recent imaging scan (x-ray, bone scan, CT, MRI, or positron emission tomography (PET) allowed), and clinically corresponding baseline bone pain of ≥ 3 points on the pain NRS. Patients with multiple radiographic and clinically symptomatic BMs sites were eligible, and patients could indeed be included for different anatomic locations. Additionally, patients needed to be able and willing to complete regular numeric pain assessments. Study exclusion criteria were MR incompatibility, radiographic or clinical evidence of pathologic fracture at the treatment site, intolerability of treatment positioning, inability or unwillingness to follow verbal commands during planning and delivery procedures as well as to grasp nature, scope and possible consequences of participating in the study.

### Screening, enrollment and study population

Metastatic cancer patients were screened for study eligibility during clinical routine, i.e., during inpatient oncology ward rounds, after direct external patient referral for palliative RT at our department or during multidisciplinary tumor boards. After a first eligibility check based on cancer diagnosis, medical history, and imaging findings, the patient was clinically assessed during a pre-treatment consultation at our department. Symptom history, pain severity per pain NRS, pain medication, and concomitant medication were protocolled. Patients were physically examined, incl. a neuro-status, depending on the site of pain. Vital signs, weight, height, and Eastern Cooperative Oncology Group (ECOG) performance status were recorded as well. If the patient remained eligible after in-consultation check, the patient was informed about the option to participate in this study, and MRI compatibility was evaluated. Upon written informed consent by the patient, the study workflow was initiated.

### Same-day MRg SF-SBRT workflow

**Fig. 1:**
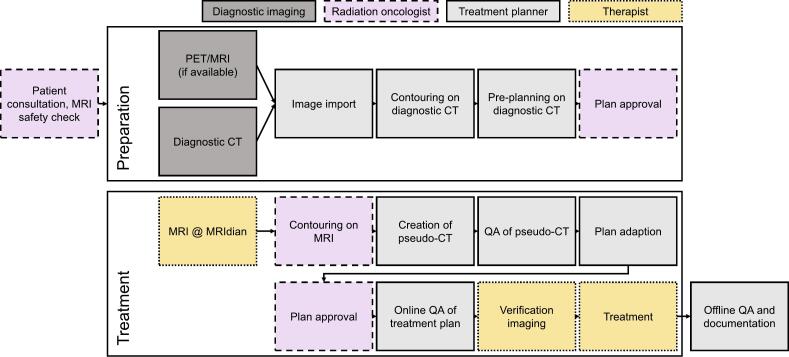
Workflow for Same-Day Magnetic Resonance-Guided Single-Fraction Stereotactic Body Radiation Therapy. Abbreviations: CT = Computer tomography; MRI = Magnetic resonance imaging; MRIdian = Hybrid MR-Linac by ViewRay; QA = Quality assurance.

The same-day MRg SF-SBRT workflow can be divided in two steps; preparation and treatment (see Fig. 1). In the preparation step, without the patient present, available diagnostic imaging data was imported into the Viewray treatment planning software (ViewRay, Inc., Oakwood Village, OH, USA, version 5.2.5.14). The gross tumor volume (GTV) and OARs were contoured by a member of the treatment planning team, with guidance from a radiation oncologist, a 5-mm safety margin was used for generation of the planning target volume (PTV) and a dummy plan was prepared on the diagnostic CT. A homogeneous dose of 12 or 16 Gy was prescribed to the PTV. (See Fig. 3a, Fig. 3b).

**Fig. 2:**
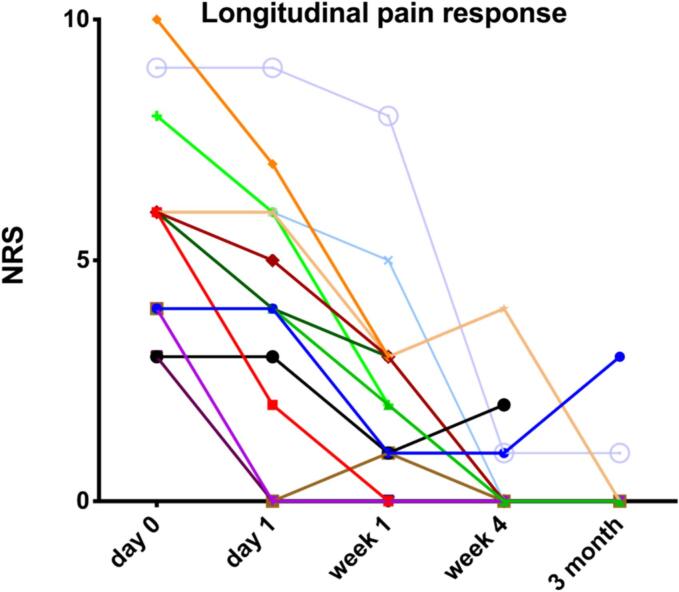
Pain response over time. Abbreviations: NRS = Numeric rating scale.Notes: Pain classified on 10-point NRS, with 0 = “no pain” and 10 = “Worst imaginable pain”.

**Fig. 3a:**
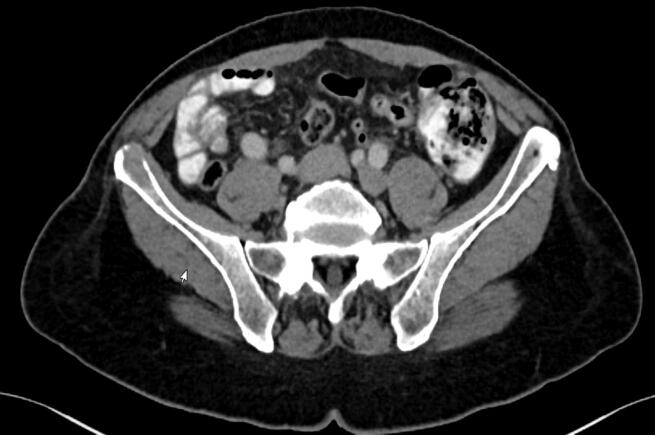
Example case – Diagnostic, axial CT image for “dummy treatment plan” creation. Abbreviations: CT = Computer tomography.Notes: Based on a diagnostic CT image, the treating radiation oncologist indicated the lesion to treat; treatment planners developed a dummy treatment plan.

**Fig. 3b:**
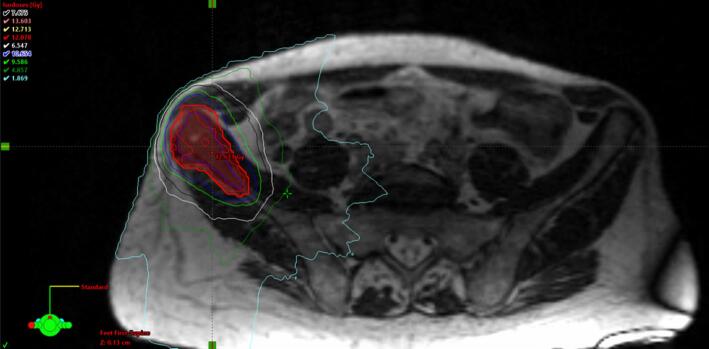
Example case – Axial slice of treatment plan of 12 Gy SF-SBRT of left hip lesion. Abbreviations: CT = Computer tomography; SF = Single fraction; SBRT = Stereotactic body radiation therapy. Notes: At the time of on-table treatment simulation, the CT-based dummy treatment plan was improved before treatment was approved by radiation oncologist and medical physicist.

The treatment was performed with the MRIdian hybrid MR-Linac (ViewRay, Inc., Oakwood Village, OH, USA). During the treatment step, the patient was present and lying in treatment position throughout the process. First, a simulation MRI was acquired. The steady-state free precession (bSSFP) sequence, resulting in a T2/T1-weighted contrast, was used. Subsequently, the radiation oncologist manually adapted the GTV, bony structures and relevant OARs based on this new MRI. The treatment planner segmented fat, air, lung and soft tissues with the aid of thresholding tools. Electron densities, as defined in the International Commission on Radiation Units and Measurements (ICRU) Report 46, were assigned to these structures to generate a pseudo-CT. The adequacy of the pseudo-CT for dose calculation was checked by re-calculating the dummy plan on the pseudo-CT and comparing it to the calculation on the diagnostic CT. Subsequently, an online adaption of the dummy plan was performed to fit the current targets and OARs. After plan approval by the radiation oncologist, the re-optimized plan was verified online with a secondary Monte Carlo calculation. Finally, a verification MRI of the patient in treatment position was acquired and the treatment was delivered. After treatment, the adapted treatment plans were verified with measurements in the Delta4 phantom (Scandidos, Uppsala, Sweden).

For the first two patients treated, the MRg SF-SBRT workflow was spread out over two days for safety and practicality reasons. In these two cases, the whole workflow including the online quality assurance (QA) was performed on day one, yet treatment was performed the following day, so that the online adapted treatment plan could be checked by a second treatment planner, and patient-specific QA could be performed before delivering treatment.

### Clinical follow-up assessment

All study patients were followed up regularly by the treating radiation oncologist for three months after treatment completion or until death, whatever event occurred earlier. Follow-up visits were scheduled at one day (D = 1), one (D = 7) and four weeks (D = 30), and three months (D = 90) (± two weeks) after completion of RT. Follow-up visits at D = 1 and D = 7 were conducted either as clinical consultation or by phone call. At every encounter, the following parameters were recorded: ECOG, pain severity according to a 10-point NRS, with 0 = “no pain” and 10 = “Worst imaginable pain”, pain medication, concomitant medication, protocol-specific adverse events, and other adverse events. Pain response as assessed according to Update of the international consensus on palliative radiotherapy endpoints for future clinical trials in bone metastases [18].

### Sample size and statistical analysis

No formal sample size calculation was done for this feasibility trial. The aim was to treat 15 NSBMs and feasibility was confirmed if > 70 % of the NSBMs were treated successfully using the same-day single session approach. As this study tested feasibility and because of the low patient number, statistical analysis remained largely descriptive and exploratory.

### Ethical approval and patient consent

This clinical study was approved by the Cantonal Ethics Committee Zurich (BASEC-Nr. 2018–01794). The study was also compliant with the current version of the Declaration of Helsinki, the ICH-GCP or ISO EN 14155 (as far as applicable) as well as all national legal and regulatory requirements. Written informed consent was obtained from all patients before study inclusion and treatment.

## Results

Between June 2019 and June 2020, 14 (100 %) patients were screened for this clinical study, 13 (92.8 %) were enrolled, one (7.2 %) excluded due to a 2-points pain score on the NRS. The 13 enrolled patients harbored 15 (100 %) symptomatic NSBMs, thus meeting the number of required interventions and making this a fully recruited trial. Median patient age was 64 (range, 30–87) years; eight patients were male (61.5 %), five patients were female (38.5 %). The most common primary tumor entities were gastrointestinal malignances (n = 5; 38.5 %), NSCLC (n = 4; 30.7 %), and prostate cancer (n = 2; 15.4 %). The majority of BMs were located in the pelvis (n = 8; 53.3 %), the second most common location was the thorax (n = 5; 33.3 %). Median baseline ECOG was 1 (range, 0–3). For clinical characteristics, see Table 1.

**Table 1:** Patient and treatment characteristics.

**Variable**	**Data (n = 13 pts; n = 15 lesions)**
Age, median (range)	64 (30–87)
Gender, n (%)	
Male	8 (61.5)
Female	5 (38.5)
Primary tumor, n (%)	
Gastrointestinal malignancy	5 (38.5)
NSCLC	4 (31.0)
Prostate cancer	2 (15.4)
Malignant melanoma	1 (7.7)
Urological cancer	1 (7.7)
Pre-SF-SBRT ECOG PS, median (range)	1 (0–3)
Treatment site, n (%)	
Pelvis	8 (53.3)
Thorax	5 (33.3)
Upper extremity	1 (6.7)
Lower extremity	1 (6.7)
Prescription dose, in Gy (n; %)	12 (13; 100 %)
GTV, median (range) in cc	15.6 (1.1.–84.4)
Dummy plan time, median (range), in min	60 (50–70)
Patient on-table time, median (range), in min	65 (57–112)

Abbreviations: ECOG PS = Eastern Cooperative Oncology Group performance status; GTV = Gross tumor volume; NSCLC = Non-small cell lung cancer; Pts = Patients; SF-SBRT = Single-fraction stereotactic body radiotherapy.

All NSBMs were treated as per study protocol; the study drop-out rate was 0 %, workflow completion rate 100 %, compared to > 70 % per study hypothesis. Eleven (84.6 %) patients with 13 (86.7 %) treated lesions completed the same-day single-session MRg SF-SBRT workflow; the first two (15.4 %) patients with two (13.3 %) lesions were subjected to a two-day schedule to verify treatment feasibility and safety for the following patients. Dummy treatment plan creation required median 60 (range, 50–70) minutes; median on-table time for re-contouring, re-planning and SBRT delivery was 65 (range, 57–112) minutes. No workflow or treatment interruption was observed for any patient.

At D = 1 and D = 7 follow-up time points, 100 % of treated patients were alive; at D = 30 and D = 90 follow-up time points, 12 (92.2 %) and 8 (61.5 %) patients were still alive; no death event was related to the palliative SF-SBRT delivered in the context of this study. No treatment-related toxicity grade 2 or higher was observed during treatment or over the follow-up period. Two patients (n = 2/n = 13; 15.4 %) reported fatigue, one at D = 1 and the other at D = 7; one patient (n = 1/n = 13; 7.7 %) reported “pain flair” at D = 7 and D = 30 follow-up time points; one patient (n = 1/n = 13; 7.7 %) reported a dermatitis at D = 90; and one (n = 1/n = 13; 7.7 %) patient reported alopecia at D = 90 follow-up time point. An overview of observed toxicities is shown in Table 2.

**Table 2:** Treatment toxicity.

**Variable**	**Data (n = 13 patients; n = 15 lesions)**
Toxicity, n (%)	
Grade 1	
Fatigue	2 (15.4)
Pain flair	1 (7.7)
Dermatitis	1 (7.7)
Alopecia	1 (7.7)
Grade 2 and ≥ 3	0 (0)

At baseline, all patients took some form of non-opioid pain medication (e.g., nonsteroidal anti-inflammatory drugs (NSARs) or paracetamol), and six (46.2 %) patients were on a daily opioid medication schedule. At baseline, 77.0 % (n = 10/n = 13) of patients took some type of non-analgetic medications in addition pain killers. Five patients (38.4 %) received a *peri*-therapeutic dose of dexamethasone at the discretion of the treating radiation oncologist. The median pre-treatment pain score on the NRS was 6 (range, 3–10) points. Median pain score on the NRS was 4 (range, 0–10) points at D = 1, 2 (range, 0–8) points at D = 7, 0 (range, 0–4) points at D = 30, and 0 (range, 0–1) points at D = 90. The reduction of the median pre-treatment pain score of 6 points to 0 points at D = 30 was statistically significant (p = 0.0028).

At D = 1, treatment response in 13 (86.7 %) lesions was indeterminate, while two lesions (13.3 %) already showed a PR. At the D = 7 follow-up time point, eight (53.3 %) lesions showed a PR, while three (20.0 %) already manifested a CR; while pain response was indeterminate in three (20.0 %) lesions, one patient (6.7 %) had pain progression (“pain flair”). At D = 30, CR, PR, and pain progression, was present in eight (53.3 %), two (13.3 %), and one (6.7 %) lesion(s), respectively; treatment response in four (26.7 %) lesions was deemed indeterminate. At D = 90, a CR persisted in six (40.0 %) lesions, a PR in one (6.7 %) lesion; no patient showed a pain progression, and treatment response in eight (53.3 %) lesions remained indeterminate. Consequently, the overall response rate at D = 7, D = 30, and D = 90 was 73.3 %, 66.7 %, and 46.6 %, respectively. For details on treatment response and pain scores, see Table 3 and Fig. 2.

**Table 3:** Treatment efficacy.

**Variable**	**Data (n = 13 pts; n = 15 lesions)**				
Treatment response, n (%)	D = 1	D = 7	D = 30		D = 90
Complete response	0 (0)	3 (20.0)	8 (53.3)		6 (40.0)
Partial response	2 (13.3)	8 (53.3)	2 (13.3)		1 (6.7)
Pain progression	0 (0)	1 (6.7)	1 (6.7)		0 (0)
Indeterminate	13 (86.7)	3 (20.0)	4 (26.7)		8 (53.3)
Pain score per NRS	D = 0	D = 1	D = 7	D = 30	D = 90
Median (range)	6 (3–10)	4 (0–10)	2 (0–8)	0 (0–4)	0 (0–1)
Pain medication, n (%)					
Baseline non-opioid	13 (100)				
Baseline opioid	6 (46.2)				
Non-analgetic medication at baseline, n (%)	10 (77.0)				
Peri-therapeutic dexamethasone, n (%)	5 (38.4)				

Abbreviations: NRS = Numeric rating scale; Pts = Patients.

## Discussion

This study enrolled 13 out of 14 screened patients with 15 NSBMs, in line with the study recruitment target. Feasibility of the same-day single session MRg SF-SBRT was demonstrated, with a 100 % workflow completion rate and a median total workflow completion time of 125 min, including preplanning and online adaptive SBRT. SF-SBRT was well-tolerated, with no treatment interruptions and no grade ≥ 2 toxicities observed. Pain per NRS at the treated site significantly decreased post-treatment, with overall and complete pain response rates of 73.3 % and 20.0 % at D = 7, and 66.7 % and 53.3 % at D = 30, respectively.

The present clinical study contributes to the expanding body of literature investigating the feasibility of same-day MRg SF-SBRT for palliative indications. With a workflow completion rate of 100 % and a median total workflow completion rate of 125 min it compares favorably with other same-day CT- as well as MR-based planning and delivery workflow studies. For 47 patients undergoing same-day CT-based palliative RT for BMs, *Nelissen et al.* (2023) reported a completion rate of 100 % and a median workflow time of 85 min. While in our MR-based study median on-table time was 65 min, in this CT-based study average on-table time was 60 min [19]. *Palacios et al.* (2022) also reported a 100 % completion rate for 10 patients undergoing a MRg SF-SBRT workflow (excluding consultation) for NSCLC, which took a median time of 6.3 h compared to about 2 h in our study, yet this was a curative setting [20]. *Schiff et al.* (2023) also reported a 100 % workflow completion rate of RT at first on-table attempt when subjecting 16 patients to MRg adaptive palliative RT; the median time from simulation to start of first treatment was reported as 407 min by the authors [21]. This figure seems large, yet it is difficult to contextualize and compare it to our genuine same-day MR-Linac workflow, as patients in the cited study were planned for and treated with either a SF- or MF-RT.

The distribution of treated sites (pelvis > thorax > extremities) in this study corresponded to the epidemiology of NSBMs and the majority of studies included into the *meta*-analysis on SBRT for NSBMs by *Moraes et al.* (2023) [17]. The two lesions in the extremities were located in the acetabulum and a femur head, so positioning efforts and workflow times were not significantly different from lesions in the pelvis or thorax. Treatment tolerability was excellent in this clinical study as well as all others found in the scientific literature [17,19,21]. Not only did no patient develop ≥ 3 toxicities in this study, yet there were also no acute grade 2 toxicities, and grade 1 toxicities were limited to the occasional, expected fatigue, pain flair, dermatitis or alopecia in less than 20 % of cases. The prevalence and severity of side effects of MRg-SR-SBRT for NSBMs thus seems comparable to those observed after conventional palliative RT [4,5].

With respect to the other secondary endpoint, treatment efficacy, this study provides early evidence for rapid-onset of pain response already at day seven post-SBRT and further improved pain response at four weeks. Meta-analyses and prospective studies evaluating pain response after conventional palliative RT provide a treatment response range between 60–80 %, with complete pain response at maximum 25 % [4,5,22]. The available retrospective studies, few prospective trials and *meta*-analyses examining the use of SBRT for non-spine BMs suggest that SBRT can at least match this short-term efficacy, while potentially providing more durable pain response due to long-term local metastases control, which is not achieved with low-dose palliative RT [11]. While in widely metastatic, severely sick and polysymptomatic patients the assessment of pain response can be difficult to record or assess, the good overall and complete response rates at four weeks and three months in this clinical study might be a further indication for the sustained efficacy of SF-SBRT in pain management, yet it might also be due to the small sample size in this feasibility study, and thus requires validation in larger patient series.

This study adds to the growing body of evidence supporting the feasibility, safety and efficacy of a same-day MRg-SF-SBRT workflow for BMs. Despite successful completion and promising results, this study has several limitations: Sample size was small, limiting the generalizability of the study findings; moreover, follow-up was relatively short, precluding long-term evaluation of treatment outcomes. Future studies with larger cohorts and longer follow-up durations are therefore warranted to validate these findings. In these settings, optimal SF-SBRT dose, dose–response relationship, systematic assessment of patient convenience, treatment comfort, and QoL and ethical considerations around same-day consent and treatment deserve attention.

In conclusion, same-day MRg-SF-SBRT planning and delivery for NSBMs was feasible, safe and achieved promising pain response. Moving forward, larger prospective studies are needed to confirm the clinical and QoL benefit of this workflow in patients with NSBMs.

Declarations.

**Ethical approval:** This clinical study (“BONE SHOT”) was approved by the Cantonal Ethics Committee Zurich before study initiation (BASEC-Nr. 2018–01794). All study-related procedures were carried out in accordance with the study and ethics protocols.

**Availability of data and material:** Collected patient data are confidential and not available for publication.

**Code availability:** Not applicable for this publication.

**Contributions of authors:** All listed authors made significant contributions to this manuscript. MG, NA and STL developed the idea and conceptualized the study. All physicians recruited patients for the study. SMC, EMK and HIGS conducted data analysis and drafted the manuscript, which was critically reviewed by all co-authors before finalization and submission. The final version of the manuscript was approved by all co-authors before submission to the journal for publication.

## Funding

None.

**Prior publication:** This study was shown as an ePoster at ESTRO’s Annual Congress in 2023 in Vienna, Austria. Yet none of this data has previously been published in the peer-reviewed scientific literature.

## Declaration of competing interest

The authors declare that they have no known competing financial interests or personal relationships that could have appeared to influence the work reported in this paper.
